# The growing methodological toolkit for identifying and studying social learning and culture in non-human animals

**DOI:** 10.1098/rstb.2024.0140

**Published:** 2025-05-01

**Authors:** Andrew Whiten, Christian Rutz

**Affiliations:** ^1^ Centre for Social Learning and Cognitive Evolution, School of Psychology and Neuroscience, University of St Andrews, St Andrews, UK; ^2^ Centre for Biological Diversity, School of Biology, University of St Andrews, St Andrews, UK

**Keywords:** animal culture, bio-logging, cultural transmission, experience-weighted attraction, network-based diffusion analysis, social learning

## Abstract

There is a growing consensus that animals’ socially transmitted knowledge should be recognized when planning conservation management, but demonstrating social learning or culture can present considerable challenges, especially in the wild. Fortunately, decades of research have spawned a rich methodological toolkit for exactly this purpose. Here, we review principal approaches, including: social learning experiments; analyses of natural or experimentally seeded diffusions of novel behaviours, sometimes using specialist statistical techniques; mapping of behavioural variation across neighbouring, sympatric or captive groups, or at larger scales; and assessment of aspects of cross-generational transmission, including teaching, learning during ontogenetic development and cumulative change. Some methods reviewed were developed for captive studies, but have subsequently been adapted for application in the wild, or are useful for exploring a species’ general propensity to learn and transmit information socially. We highlight several emerging ‘rapid assessment’ approaches—including camera trapping, passive acoustic monitoring, animal-borne tags, AI-assisted data mining and computer simulations—that should prove useful in addressing particularly urgent conservation needs. We conclude by considering how best to use this growing methodological toolkit in practice, to guide further research on animal social learning and cultures, and maximize conservation and policy impact.

This article is part of the theme issue ‘Animal culture: conservation in a changing world’.

## 1. Introduction

In this article, we review methods for identifying and studying social learning and culture in non-human animals (hereafter ‘animals’), as applicable in both fundamental and applied research. We adopt the broad definition of *culture* by Laland & Hoppitt: ‘Cultures are those group-typical behavior patterns shared by members of a community that rely on socially learned and transmitted information’ [1,2]. *Traditions* may be defined in the same way, and for many researchers, the two terms are synonymous. However, once tradition has been defined as above, a community’s culture as a whole can be thought of—as in human cultures—as an *array of traditions* covering different domains of behaviour, such as migration, foraging and communication [3]. *Social learning*, central to the definition of culture, is broadly understood as learning from others,^1^ rather than learning through one’s own efforts alone, with multiple different underlying processes recognized (see §2; [2]).

Culture was once thought a uniquely human trait, but the last 70 years have seen evidence for cultural phenomena accumulate across the animal kingdom. A recent review [5] showcased culture’s reach across diverse animal species (including all main groups of vertebrates and, at least in the lab, insects), different domains of behaviour (including diet choice, foraging techniques, tool use, nest and breeding site selection, mate choice, vocal repertoires and migratory routes), an organism’s lifetime (extending to adults learning when dispersing to new ranges or groups), different types of social learning (including local enhancement, imitation and teaching) and numerous adaptive social learning biases (such as conformity to existing majority behaviours). Other recent reviews provide complementary perspectives [6–11].

Major conservation bodies have begun to take note of these discoveries and consider their practical implications. The United Nations Environment Programme’s (UNEP) Convention on the Conservation of Migratory Species of Wild Animals (CMS) established an Expert Working Group on Animal Culture and Social Complexity in 2018, leading to recommendations for conservation management and policy making [12,13] and the approval of Concerted Actions focused on chimpanzee cultures [14]. Meanwhile, groups affiliated with the International Union for Conservation of Nature (IUCN) incorporated what has been learned of cultures in wild great apes into detailed action plans for the conservation of eastern [15] and western chimpanzees [16]. CMS and IUCN are increasingly collaborating on the coordination and integration of these initiatives [14].

These and similar conservation-focused efforts clearly depend on identifying social learning and culture in the animals of interest, which can be challenging. According to the above-mentioned definition of culture, reliance on socially learned and transmitted information needs to be demonstrated. Moreover, while documenting social learning is necessary for confirming the existence of culture, it is not sufficient, because the effects of social learning may be relatively transient (such as finding an ephemeral food resource) or may otherwise fail to lead to recognizable traditions. That said, demonstrating social learning *per se*, even in contexts where effects remain transient or involve transmission from just one individual to another, can have important implications for conservation practice [12,13]. Accordingly, this article spans both social learning and the larger phenomena of traditions and culture.

Fortunately, decades of research have spawned a rich toolkit of methods for the identification and study of social learning and culture. In this article, we present a concise overview of the approaches available, to provide guidance for planning or refining studies in this area (see also [2,17]). Other contributions to this theme issue address linkages between social learning and culture, once identified, and conservation management and policy making, as do the CMS and IUCN plans mentioned above. Given insufficient space for an exhaustive review, we draw on our familiarity with primate and avian studies to illustrate key concepts, supported by electronic supplementary material, table S1, which provides an expanded set of 80 diverse applications of 14 different methodological approaches. We conclude with thoughts on how to use the toolkit, acknowledging various practical constraints.

## 2. Social learning

Social learning can occur via visual, auditory, olfactory or other modalities, and through processes of varying complexity, ranging from the learner’s attention being drawn to particular targets (stimulus enhancement) or locations (local enhancement) to the copying of hierarchically structured procedures (programme-level imitation) [18,19]. Culture requires the repeated occurrence of social learning, as behaviours diffuse across a group, and/or across generations. In an experimental context, social learning is neatly demonstrated if a novel behaviour is more likely acquired when subjects are exposed to a conspecific ‘model’ performing that behaviour, than in a control condition lacking a model. In contrast, to test for *cultural transmission*, investigations must go further, examining whether the novel behaviour spreads to other individuals, once exposed to those already proficient in it, thus creating an incipient tradition or sustaining one (§4).

Although it is important for researchers to recognize when they have evidence for social learning, but not yet for the larger phenomenon of culture, this distinction may not always be clear. Indeed, in the approach we discuss next, it is the pattern of behavioural diffusion which itself provides the principal evidence for social learning.

## 3. Tracing the spontaneous diffusion of a novel behaviour

### (a) Descriptive and quantitative records

When researchers are lucky enough to recognize that a novel behaviour has appeared, its subsequent diffusion across a population may be tracked to investigate whether it becomes established as a tradition and/or contributes to a local culture. This has often been done without the sophisticated statistical approaches described below. In Fisher & Hinde’s foundational study of British tits tearing open the tops of milk bottles left on doorsteps [20], diffusion of the behaviour could be mapped using amateur ornithologists’ reports spanning several decades. Electronic supplementary material, table S1, provides seven further examples of studies using descriptive records and simple quantification, covering birdsong and a variety of behaviours in cetaceans and primates.

### (b) Discriminating diffusion curves

Cavalli-Sforza & Feldman [21] proposed that the cumulative curve expressing the diffusion of an invention is almost always S-shaped: in the earliest phase, there are few models to learn from so the curve rises only slowly, but it subsequently accelerates as models proliferate, and finally tails off again as the number of naive individuals diminishes. In contrast, they assumed a linear pattern would indicate asocial, individual-level learning of a novel behaviour. While the prospect of identifying cultural diffusion in this way inspired valuable analyses (e.g. of 21 putative primate cases: [22]), later appraisals of the approach have noted that factors other than social learning may create a logistic or other accelerating function, or alternatively, that this pattern may not be evident even when cultural diffusion is truly at play [23]. Researchers studying the spread of a novel behaviour among unidentified individuals should note that there is now broad agreement that the shape of diffusion curves does not reliably indicate social transmission [2].

### (c) Network-based diffusion analyses

Recognizing the limitations of diffusion-curve analyses, Franz & Nunn [24] suggested that social learning can be more confidently inferred if a novel behaviour is shown to spread systematically across a network of social relationships between identified individuals—that is, with transmission occurring between individuals most closely associated with each other. Such network-based diffusion analysis (NBDA) was subsequently refined by Hoppitt *et al*. [25], distinguishing between a time of acquisition analysis (TADA), following Franz & Nunn [24], and a new version that instead focused on the order of acquisition of the behaviour of interest (OADA). Both methods were soon used to identify the diffusion via social learning of lob-tail feeding in humpback whales [26]. The behaviour, which involves smacking the tail on the water surface to corral fish for capture, spread from the earliest observations for just one or two whales to 241 out of 653 identified individuals over 26 years; importantly, NBDA analyses confirmed diffusion across the social network, based on associations between whales observed over this period, thus implicating cultural transmission. A much faster diffusion was recorded following the invention of ‘moss-sponging’ in chimpanzees [27]. Here, NBDA was modified to exploit the fact that, instead of using the proxy of social association, direct records of chimpanzees observing those already performing the behaviour were available.

NBDA analyses have since been further refined ([2,28]; in particular, see [29,30]) and applied across a growing number of species, including cockatoos and dolphins (electronic supplementary material, table S1). This powerful statistical approach could be extended to accommodate incomplete datasets—for example, where information is missing for parts of the underlying social network and/or for some cases of individuals acquiring the target behaviour (W. Hoppitt, personal communication, 2024). Such advances would enable even wider applications, including in conservation contexts where data deficiency is a common challenge.^2^

### (d) Agent-based modelling

Agent-based models (ABM) have recently been used to study social learning and culture in combination with approximate Bayesian computation (ABC), which is applied to identify the best estimates of model parameters that fit the empirically observed behaviour. This is thus a form of simulation-based or generative inference ([32,33], for reviews). Youngblood & Lahti [34] provide an example revealing an extremely high transmission fidelity (median >99.9%) of house finch song over a 44 year period. To date, this approach appears to have been applied only in studies of bird and whale song (electronic supplementary material, table S1).

## 4. Experimentally seeding potential diffusions

### (a) Methodological frameworks established in captive studies

The approaches above may be of particular relevance to the topic of this theme issue, but they all depend on the chance discovery of a spontaneous diffusion event, and such opportunities may be rare. However, valuable insights have emerged from a literature stretching back into the last century, in which the potential for cultural diffusion is experimentally created by researchers introducing models trained to perform novel behaviours into captive populations of naive individuals. For many species of conservation concern, such controlled experiments may be possible in captive breeding facilities, to inform the reintroduction or management of wild populations.

Whiten & Mesoudi [35] reviewed 27 diffusion studies with animals for 1972−2008, and Whiten *et al*. [36] identified an additional 30, with 23 and 16 of these coming from captive populations, respectively. Three main experimental designs were distinguished, each of which has counterparts in nature: ‘open group’, in which a model or models performing a novel behaviour is introduced into a population that is open with regard to who observes whom, and which individuals, if any, acquire the seeded behaviour; ‘replacement’, in which some individuals are repeatedly replaced by new, naive individuals; and ‘transmission chains’, in which the first individual to learn from the model becomes a model for another, and so on, along a potential chain of ‘cultural generations’. In addition, seven conditions were differentiated by the presence or absence of no-model control groups to determine the likelihood of invention of the behaviour in question by naive animals, and the inclusion or not of ‘two-action’ (or ‘two-option’) designs in which two or more models are trained to display different forms of behaviour in their respective groups. Of the 21 cells in the resulting matrix of three experimental designs by seven conditions, 15 were represented by published studies (both captive and wild).

In one example, a first group of chimpanzees was seeded with a two-step procedure to extract food rewards from a large ‘artificial fruit’, while a second group was seeded with a different pair of procedures, before which a no-model baseline condition showed that no chimpanzees managed to extract a food reward through their own personal efforts [37]. A further two groups acted as no-model controls for a similar period as the seeded groups, with a similar result. In contrast, the alternative procedures spread in the groups they were seeded in (an example of open diffusion), and each procedure was then transmitted to a second and a third group in a group-chain design. Bandini *et al*. [38] discuss what can be learned from different experimental and control conditions in studies investigating the development of tool-related behaviours, but their considerations apply more broadly to cases where the challenge is to determine the relative contributions of social learning, individual learning and genetic predispositions to the acquisition of specific behaviours. These considerations are relevant to recent debates about distinguishing cultural transmission from independent reinventions [39–42].

### (b) Experiments in the wild

Just 18 of the 57 diffusion experiments noted above were conducted in the wild [35,36]. Working with wild animals entails many additional challenges—including training models without group members seeing this, and testing naive individuals alone in no-model control conditions (and for important ethical considerations, see §8). In a notable avian example, pairs of wild great tits were captured from different populations and trained to push the blue side of a feeder cover to the right, or the pink side to the left, to gain a food reward. They were then released back into their respective wild populations where they could act as models for others [43]. Each of the two alternative feeding techniques spread with remarkable fidelity across social networks identified through NBDA within the models’ local populations. A high fidelity of transmission was further maintained into the following season, as new recruits joined.

In a different approach, rather than training any models, Canteloup *et al*. [44] provided different groups of wild vervet monkeys with eight artificial fruit boxes that could be opened in either of two different ways, but left it to the boldest monkeys to discover how to succeed. Luckily, the highest-ranking female in one group was the first to open the box using one technique, while the highest-ranking male in the other group was the first to use the alternative technique, resulting in a natural two-action experiment. Application of the OADA type of NBDA provided strong evidence for social transmission. The approach in this experiment—providing an artificial foraging device that affords different techniques rather than training models—could be particularly informative in that it allows assessment of which specific novel behaviours spark social transmission (see also [45]).

While field experiments have continued to proliferate, our focus here is on outlining the different methods employed. Variant designs have investigated: the adaptive flexibility of asocial and social learning when a current tradition becomes unrewarding and a new opportunity is available to be discovered [46]; the prospects for mastering cumulative complexity in foraging challenges; the role of demographic factors, such as population turnover; and the effects of training whole groups to express different behavioural variants (electronic supplementary material, table S1). These field studies are also revealing limitations on cultural transmission through social factors such as competition, and learning processes such as generalization [45], with implications for charting the potential utility of these phenomena for guiding conservation management.

### (c) Incorporating experience-weighted attraction models

Experience-weighted attraction (EWA) models are designed to use sequential data on individuals’ behaviour to discriminate the effects of both social and asocial experiences [47,48]. For example, social input may correspond to what the target animal observed another individual do before acting itself, and asocial input to their accumulated asocial experiences, such as rewards obtained. In this way, Aplin *et al*. [46] identified both conformist learning and learning from others’ observed payoffs in a foraging context. The method has similarly identified social learning in the simplest kinds of social learning experiments, involving the mere introduction of a novel food, where an asocial control condition may be difficult or impossible (electronic supplementary material, table S1)—a common constraint in the wild [49]. All four additional examples in electronic supplementary material, table S1, illustrate methodological advances.

## 5. Identifying stable inter-group cultural variation

Relatively few studies have been able to record spontaneous instances of novel behaviours diffusing across natural populations (§3). Many socially transmitted behaviour patterns appear relatively stable over the lives of the animals under investigation, as well as the careers of the researchers and conservation practitioners who study them, and experimental manipulations (§4) may be either impractical or deemed unethical (§8). An alternative approach in these circumstances has been through recording variation in behaviour, or behavioural repertoires, between groups or wider populations.

### (a) Broad geographic variation

Striking patterns of behavioural variation have been documented across large geographic areas in various primates, cetaceans and birds. For example, differences have been documented in 22–39 foraging, tool use and social behaviour patterns in chimpanzees, gorillas and orangutans (electronic supplementary material, table S1). This approach came to be called the ‘method of exclusion’, because culture was inferred so long as research teams were confident in ruling out ecological and genetic explanations for observed differences in behaviour.^3^

These pioneering efforts stimulated many further studies of animal cultures, but limitations inherent in the approach have sparked lively debate that potential users should be aware of [52–54]. Most fundamentally, it is important to recognize that establishing behavioural differences between populations is, in itself, insufficient to infer a cultural basis. The development of behaviour typically results from a complex interplay of genetics, social learning and asocial learning, modulated by prevailing environmental conditions. In attempting to rule out ecological and genetic explanations, the method of exclusion faces two (opposing) limitations. On the one hand, it may *under-estimate* the scale of culture, because it relies on excluding ecological explanations for behavioural variation, ignoring the fact that culture is often locally adaptive [50]—as evident in human culture, such as the subsistence technologies adapted to harsh environments. On the other hand, the approach may *over-represent* the scale of culture, because cases can be erroneously included where genetic factors or local environments shape behaviour via asocial learning [52,53].

Some researchers have thus gone on to include data on relevant ecological and/or genetic factors in their analyses, both for primates [55,56] and cetaceans [57]. However, even then, we must acknowledge that it is conceptually impossible to prove the absence of a cause [52].^4^ In the end, expert judgement must be used to interpret the available evidence, and the scientific community will assess how compelling particular inferences are. For example, Boesch *et al*. [56] recently reported differences in combinations of behavioural techniques, postures and tool types used by chimpanzees to fish for subterranean termites at six different study sites in Central Africa. Parameters such as termite nest depth and structure could not explain the observed differences in chimpanzee behaviour, leading the authors to conclude that they had discovered unexpected cultural diversity.

Reflecting on the above, we see the mapping of between-group behavioural variation as a valuable starting point for formulating hypotheses about cultural candidates, prompting further investigation with other methods that seek to assess the relative contributions of different underlying processes [59]. As noted previously, controlled experiments, in the field or laboratory, provide a powerful means for establishing causality. Thus, in chimpanzees and orangutans, application of the method of exclusion led to confirmatory diffusion experiments that demonstrated the species’ capacity to transmit alternative forms of tool use and other actions they witnessed [37,60]. In the case of chimpanzees, multiple different types of evidence (as surveyed in this article) have accumulated over time, providing diverse but convergent evidence supportive of earlier cultural interpretations (§8). In a contrasting example, claims of material culture in tool-using New Caledonian crows, based solely on spatial variation in tool designs [61], remain premature, since social learning has not been demonstrated for the tool type of interest [59] (§6e).

In many cases, the interpretation of (large-scale) patterns of behavioural variation can be informed by specific knowledge of how the trait of interest is being transmitted (§3). For example, controlled experiments show that bird song has a socially learned component in numerous species [62], supporting the interpretation of regional and changing dialects in the wild as cultural ([63] for a review). However, experiments are not the only kind of valuable supporting evidence: the speed of diffusion in some cases of cetacean song, for example, is so fast as to leave only cultural processes as a plausible explanation [64,65].

### (b) Variation between neighbouring or sympatric groups in the wild

A complementary approach is to compare directly neighbouring groups or populations, which share a similar habitat and experience gene transfer between them, allowing stronger inferences about a role for culture. In this way, cultural differences among three neighbouring groups of chimpanzees in their seasonal use of wooden and stone hammers to crack open nuts have been evidenced [66], with additional examples for chimpanzee gestures and vervet monkey social dynamics. Habitat differences are even more clearly controlled for when the comparison is focused on areas where the ranges of animals overlap. For example, communities of bonobo have been shown to have quite different prey preferences during their hunts in a large, shared area, and there is notable variation in the vocal repertoires of sympatric clans of sperm whales. Some studies additionally report evidence of transmission between neighbouring groups. References for these diverse studies are provided in electronic supplementary material, table S1.

### (c) Variation between captive groups

Studies exploring behavioural variation across captive groups deserve mention because they can often best control genetic and environmental factors, and illustrate the cultural capacities of the species concerned when field observations are still inconclusive. Such work may be possible with endangered species held in zoological gardens, rescue centres, or dedicated conservation breeding facilities. For example, multiple behavioural differences have been recorded among four neighbouring chimpanzee sanctuary enclosures in Zambia, including the habit of placing and leaving a stem of grass in one ear, which spread to most of the single group in which its initiation was recorded [67]. Similarly, hand-clasp grooming occurred in only two groups, and groups also differed in the techniques used to crack open hard-shelled fruits (electronic supplementary material, table S1). These patterns mirror on a small scale those observed in the wild (§5a).

## 6. Focusing on cross-generation transmission

### (a) Tracing transmission from informed to naive individuals

Goodall [68] described how young chimpanzees showed avid attention to their mothers’ tool use, and interpreted this as social learning. However, youngsters may be motivated to attend closely so as to scrounge morsels, and resemblance between mothers and offspring could be due to genetic factors—which might explain, for example, the finding of Jaeggi *et al*. [69] that young orangutans’ diets were virtually identical to their mothers’, despite great variation between maternal dietary profiles. More compelling evidence that social learning may be implicated comes from the fact that youngsters attended preferentially to their mothers’ expertise in difficult extractive foraging tasks [70].

Building on this work, Schuppli *et al*. [71] tested five predictions derived from the hypothesis that close peering by juveniles has a learning function. All five were confirmed, such as that, after watching an activity (such as food processing, tool use or nest building), the youngster spent the next hour on the same activity. Having obtained such confirmatory evidence, Schuppli & van Schaik [50] went on to document as many as 195 different contexts in which such peering occurred during the development of youngsters in the wild. Interestingly, as male juveniles matured and later dispersed, they devoted relatively more attention to the foraging activities of adult males, consistent with the hypothesis that they gradually shifted to learning about the males’ significantly different foraging niche [72,73]. Sex differences in peering have also been identified in young chimpanzees learning termite fishing [74], and additional examples are provided in electronic supplementary material, table S1.

A different contrast was explored in a study of banded mongooses, in which youngsters form close associations with providers known as ‘escorts’—usually, young non-breeding adult males. Exploiting the fact that these animals crack open hard-shelled items, preferentially by either holding them and biting them open, or smashing them on a hard surface, escorts of each type were provided with artificial objects that could be cracked open to reveal food. Youngsters tended to adopt the technique demonstrated by their escorts, suggesting that these techniques are socially transmitted [75].

### (b) Cross-fostering

Cross-fostering directly tests whether young will learn from foster parents who behave differently to their biological parents. This is more practical in birds than in mammals, because eggs can be swapped relatively easily, as was done between the nests of sympatric great tits and blue tits [76]. This study demonstrated that the young birds acquired aspects of the behaviour typical of their foster parents (rather than their genetic parents), such as preferred foraging height in the canopy. While such cross-fostering can provide compelling experimental evidence for social learning, we caution that the ethics of conducting such manipulations in wild populations must be evaluated carefully. In range-restricted or endangered species, we suggest such work is not advisable—for example, to shed light on population-level differences in New Caledonian crows’ tool behaviour [59].

### (c) Investment in transmission: teaching, functionally defined

The bulk of the methods and studies reviewed in this article are focused on social transmission that occurs when individuals learn from conspecifics that are essentially going about their normal business, or from the products they leave behind, such as discarded tools, constructions or food items.^1^ In teaching, on the other hand, the individual who serves as a model plays an active part in the process. Animal studies typically follow a definition that requires that a teacher acts in some way to support skill development in a novice animal, at some immediate cost to itself. This is a functional definition, avoiding reference to any intent to teach, which is often assumed in the human case. A classic example is adult meerkats initially removing the sting of scorpions before presenting them to pups, who practice despatching prey [77]. Later on, when pups have developed appropriate competence, scorpions with intact stings are provided, and retrieved if they escape. Candidate cases of teaching come from diverse taxa, including various mammals, birds and insects [78–81] (electronic supplementary material, table S1). Where such behaviours can be recognized in the species of interest, further investigations into potential traditions or cultures are merited.

### (d) Archaeology

Archaeological digs have shown that nut cracking using stone hammers has existed for over 4000 years in chimpanzees [82] and over 3000 years in capuchin monkeys [83]. Searching for archaeological evidence of culture may seem a niche approach, but the systematic analysis of tools and other artefacts left behind by animals over shorter timescales can be a productive avenue [61,84], although for perishable items, repeated sampling over longer periods is required for investigations of cross-generational transmission.^5^

### (e) Cumulative change over generations

Cumulative culture, or *cumulative cultural evolution* (CCE), involves the repeated incorporation of innovations into a population’s culture such that greater efficiency and/or complexity is achieved [86–88]. Human culture is marked by unprecedented forms of CCE [89], but tentative evidence has begun to emerge for other animals [90].

Bighorn sheep, which track the availability of graze at progressively higher altitudes during their spring migration, offer an example. After some populations were extirpated by humans in past centuries, translocations were later conducted to replace them (for the role of culture in translocation efforts, see Greggor *et al*. [91]). Initially, no migration occurred, but over decades and generations, not only did seasonal migration continually extend, but individuals became progressively more skilled at arriving at higher patches of graze at the optimal time [92]. The authors contend that this could have occurred only through CCE, with within-generation improvements being cumulatively incorporated into the populations’ culture.

An often-cited example of a possible case of cumulative *material* culture is the production of different tool designs from screw pine leaves (*Pandanus* spp.) by New Caledonian crows [61]. While the degree of technological diversification in this species is remarkable [93], on current evidence, the relative contribution of social learning to the behavioural differences observed between populations remains unclear [59,94]. Uncertainty about the transmission mechanism aside, the best evidence to date for progressive, efficiency-enhancing design modifications comes from another tool type used by some New Caledonian crow populations—stick tools. Hooked stick tools, which have a small, crow-made hook at the functional tip [93], have been shown to be functionally superior to non-hooked tools, in controlled experiments with wild-caught subjects [95]. Importantly, some hooked stick tools exhibit additional crow-made design features that may further enhance efficiency (a bent tool shaft and stripped bark at the tip), illustrating how design innovations may accumulate over time [96]. While one of the most compelling cases of cumulative technological refinement in a non-human species [94], further evidence is required to demonstrate *cultural* transmission and, hence, CCE.

Changes in some avian vocal repertoires have now been charted across decades [97]. Their interpretation as CCE is controversial in terms of progressive increases in efficiency and/or complexity [63,98], but they certainly provide compelling examples of cultural *change*. In the case of humpback whales, there is evidence of progressive rises in song complexity, followed by falls when ‘revolutionary’ new songs emerge [99].

## 7. Ancillary techniques

### (a) Technology-driven methods

All the methods discussed above rely on documenting the behaviour of captive or wild animals, commonly with a focus on distinct behavioural variants—such as different types of vocalization or foraging techniques. In an ideal case, individuals are identifiable through natural or researcher-deployed markers, enabling the assignment of specific behaviours or behavioural repertoires, or the tracing of the diffusion of novel behaviours across social networks. The last few decades have seen an explosion of technological advances facilitating the direct observation of, or indirect inferences about, animal behaviour across scales, from individuals to populations, considerably enriching our toolkit. Here, we briefly review some particularly useful ancillary methods.

The use of camera traps and passive acoustic monitoring systems is now widespread [100], allowing researchers not only to confirm the presence of species in particular habitats, but also to chart animals’ behavioural and communication activities (see below). The principal strength of these technologies is that data collection is autonomous (notwithstanding the need to service field-deployed units), non-invasive (animals do not need to be trapped and handled) and scalable (arrays of recorders can be used to cover large study areas). However, given that cameras and/or microphones are typically stationary, their physical range for data collection is limited (drones offer an alternative for mobile surveillance), and when fitted with basic trigger mechanisms, vast amounts of data may be captured, yet fail to document the behaviours of interest.

A complementary approach is the deployment of miniature electronic tags on the animals themselves, to collect—via various sensors—data on their movements, behaviour, physiology and/or environments. Over the past few decades, such ‘bio-logging’ has revolutionized wildlife research and conservation in both the terrestrial and marine realms [101,102]. For addressing the specific challenge of detecting variation in behaviour that could be indicative of cultural processes, tri-axial accelerometers (and magnetometers) seem particularly promising. These sensors, which are contained in most human ‘wearables’ like smart watches, produce records of body motion and orientation that can be used to estimate activity budgets [103]. Pioneering studies have used onboard (AI-assisted) processing to detect rare target behaviours in accelerometer data, to then trigger video cameras for further data collection [104,105]. Alternatively, animal-borne tags can guide drones, to closely monitor specific animals and their environments [106]. Some of the most compelling demonstrations to date of the conservation-relevance of socially learned behaviours have been achieved with bio-logging technology, including the mapping of large-scale migratory movements [92,107]. The application range of bio-logging is vast, but it is paramount to carefully assess, on a case-by-case basis, the ethics of tagging wild animals [108].

Several lab-based methods can generate valuable complementary data. Stable-isotope profiling, for example, is a well-established method for assessing animals’ dietary preferences from small blood, skin or feather samples, enabling efficient investigation of potential differences between groups or changes over time [109]. Where taxonomic identification is required, and samples of food remains, faeces or even discarded plant tools are available, DNA barcoding may offer a solution [110]. More generally, genetic (and comparative genomic) methods can be used to establish the identities and relationships of unmarked individuals, to chart population structure and divergence [111], and increasingly, to search for genomic correlates of specific behaviours [112].

The advent of machine learning (ML) is fast transforming data collection and analysis efforts in the fields of animal behaviour and conservation [113]. ML is ideally suited for processing vast volumes of images, video footage or audio recordings—running various detection, tracking and classification tasks with minimal human supervision (e.g. for application to animal communication, see [114]). For example, to aid research on wild chimpanzees, ML tools have been developed for: identifying individuals from their faces alone [115]; detecting behaviours of interest, such as buttress drumming or nut cracking [116]; providing automated pose estimation [117]; and classifying vocalizations [118]. Given the current interest in ML, we expect significant investment over the coming years into further methodological refinement, the development of new tools, and application across a wide range of study systems and contexts. That said, given the non-trivial environmental footprint of these computationally intensive approaches, we urge the community to carefully assess the costs and benefits of studies, and use traditional, minimal-impact alternatives where possible.

Large-scale, collaborative work can enable relatively rapid assessments of behavioural variation, supporting analyses of group-, population- and even species-level patterns. For example, the ‘Pan-Af’ project maps chimpanzee behavioural diversity in a large number of replicate sites across the species’ entire range (limiting work at most sites to 12 months), using techniques such as camera traps trained on strategic locations, like termite mounds [119]. While this approach inevitably yields only a partial record of local behaviour, and has limited power to confirm the absence of behaviours that are known to be common elsewhere (cf. [51]), it has generated a wealth of new information. Findings with important conservation implications include demonstrations of the impact of human disturbance on behavioural (and presumed cultural) diversity [119,120] and of fine-scale differences in specific forms of tool use that appear explicable only as cultural variants [56,121]. In some study systems, it may be possible to assess social population structure over larger areas by using a readily surveyable proxy, such as vocalizations; once social units have been identified, targeted follow-on work can focus on other behaviours, such as specific foraging techniques, which are usually more challenging to study (crows: [122]; orcas: [123]).

Although we see huge potential for these technology-driven methods in this field, to support the collection and analysis of increasingly large volumes of detailed data, we note that they cannot by themselves demonstrate the existence of social learning or culture. They can neither replace in-depth knowledge of a species’ natural history, nor the need for carefully designed experiments or observational studies.

### (b) Computer simulations

Opportunities to use *in silico* tools in this field are proliferating. Analyses can not only aid assessments of whether (or not) observed behaviours are socially learned (§3), but may also be used to *predict* the potential reach and significance of cultural processes. For example, for a population of New Caledonian crows, proximity logger-recorded dynamic social networks were used to simulate the diffusion of information within and between multi-family communities, to inform discussions about the putative role of culture in this prolific tool user ([124]; §6e).

Despite growing recognition that animals’ social lives should be considered in conservation planning [12,13], how best to achieve this in practice remains to be worked out for most study systems. We suggest the time is ripe to go beyond intuitive assessments and narrative accounts by leveraging existing empirical data to parameterize computer models that allow formal exploration of key issues (cf. §3). For example, we envision *in silico* assessments of the potential fitness benefits of socially transmitted behaviours, or of the effectiveness of different conservation interventions. As in more mature fields of interdisciplinary collaboration, this could create a productive feedback loop between theoretical and empirical research, significantly accelerating progress towards formalizing conceptual frameworks and formulating specific management and policy recommendations—both of which are urgently needed at this stage.

## 8. Concluding remarks

We conclude with some thoughts on how best to use the methodological toolkit in practice. First of all, it is important to recognize that the approaches outlined above yield evidence of varying strength. In general, an experiment is expected to produce more compelling evidence of social learning than an observational study, and replication—across groups of animals, geographic locations, different study contexts, and/or research teams—can serve to strengthen the overall weight of evidence (the STRANGE framework for animal behaviour research may help mitigate sampling biases; [125]). Second, we reiterate the importance of carefully assessing the ethical implications of potential methods. For example, although experiments may promise strong results with relatively little effort, they may not be an acceptable choice for systems where they could cause harmful impacts—for example, by altering natural behavioural repertoires in small populations. Finally, there are obvious practical constraints: for example, with some species, it is simply impossible to set up well-controlled diffusion experiments, while with others, cutting-edge bio-logging approaches remain out of reach because tags cannot be safely attached.

The production of multiple, convergent lines of evidence in a given study system can be particularly compelling. For example, at least seven different methods provide evidence that tool use in chimpanzees is under the influence of cultural processes [126], arguably making this the best-documented case of non-human culture. In fact, it was this body of evidence that created an opportunity for a conservation-focused policy intervention, via a CMS Concerted Action [12,14]. However, gathering such data takes time: the initial systematic analysis of behavioural variation in wild chimpanzees alone was based on a total of 151 years of observations across study sites [51]. Accordingly, we have highlighted some opportunities above for accelerating data collection and analyses with various ancillary methods, which may be particularly helpful for informing urgent work on endangered species (§7). More generally, we advocate a pragmatic approach, acknowledging the fact that the bar for demonstrating culture may be higher in a pure-science academic study, than in a conservation project where a time-sensitive management decision is required, and the precautionary principle may reasonably be applied. The methodological toolkit for identifying and studying animal social learning and culture is now sufficiently well-developed to support a broad range of applications [14,65,91,127], explored throughout the remainder of this theme issue.

## Data Availability

Supplementary material is available online [128].

## Footnotes

^1^
A more comprehensive definition of social learning was provided by Heyes [4], identifying it as ‘learning that is influenced by observation of, or interaction with, another animal (typically a conspecific) or its products’ (p. 207). This is helpful in highlighting that an animal may learn from others in respect of both their behaviour and its *results* (e.g. a discarded tool). However, since ‘observation’ may risk neglecting learning based on modalities other than vision, we refer here simply to ‘learning from others’.

^2^
Even when the diffusion of a novel behaviour has not been witnessed, time-ordered data of known (or likely) social contacts between animals can be used to estimate the minimum number of independent invention events needed to produce—via social transmission—the behaviour’s observed, contemporary distribution. This approach can be employed to gauge the likelihood, and potential pathways, of cultural diffusion [31], potentially enabling rapid assessments to inform conservation action.

^3^
Identifying culture through group differences has sometimes been misinterpreted as a necessary part of the *definition* of culture. This was not intended by the foundational studies, which purely sought to chart potential cultural variation in the wild. There is no reason why a behaviour cannot be widespread in the species of interest, but also be part of its culture, because it is socially transmitted [50,51].

^4^
In one of the 39 behavioural variants reported in the initial chimpanzee studies [51], where the difference involved using long versus short sticks to dip for ants, similar tool selectivity was later observed at a single site according to whether the targeted ants had a mild or severe bite [58]. However, no such cautionary case has arisen for the other cultural candidates.

^5^
Work in this field revealed, for example, variation in the tools discarded by neighbouring chimpanzee communities [85] and the shape of leaf tools produced by New Caledonian crows [61]—although, as noted in the main text, such evidence alone is insufficient to demonstrate the existence of cultures. A promising avenue is, in our view, the assessment of time series of artefactual records, such as tool designs, to detect signatures of behavioural divergence or rapid diffusion that may be indicative of cultural processes.

## References

[1] Laland KN, Hoppitt WJE. 2003 Do animals have culture? Evol. Anthropol. **12**, 150–159. (10.1002/evan.10111)

[2] Hoppitt W, Laland KN. 2013 Social learning: an introduction to mechanisms, methods, and models. Princeton, NJ: Princeton University Press. (10.23943/princeton/9780691150703.001.0001)

[3] Whiten A, van Schaik CP. 2007 The evolution of animal ‘cultures’ and social intelligence. Phil. Trans. R. Soc. B **362**, 603–620. (10.1098/rstb.2006.1998) 17255007 PMC2346520

[4] Heyes CM. 1994 Social learning in animals: categories and mechanisms. Biol. Rev. **69**, 207–231. (10.1111/j.1469-185x.1994.tb01506.x) 8054445

[5] Whiten A. 2021 The burgeoning reach of animal culture. Science **372**, eabe6514. (10.1126/science.abe6514) 33795431

[6] Allen JA. 2019 Community through culture: from insects to whales. BioEssays **41**, e1900060. (10.1002/bies.201900060) 31631360

[7] Aplin LM. 2019 Culture and cultural evolution in birds: a review of the evidence. Anim. Behav. **147**, 179–187. (10.1016/j.anbehav.2018.05.001)

[8] Duboscq J, Romano V, MacIntosh A, Sueur C. 2016 Social information transmission in animals: lessons from studies of diffusion. Front. Psychol. **7**, 1147. (10.3389/fpsyg.2016.01147) 27540368 PMC4973104

[9] Whitehead H, Rendell L. 2014 The cultural lives of whales and dolphins. Chicago, IL: University of Chicago Press.

[10] Whiten A. 2019 Cultural evolution in animals. Annu. Rev. Ecol. Evol. Syst. **50**, 27–48. (10.1146/annurev-ecolsys-110218-025040)

[11] Sanz C, Whiten A. 2024 Animal cultures. In Oxford bibliographies online. Oxford, UK: Oxford University Press. (10.1093/OBO/9780199766567-0290)

[12] Brakes P *et al*. 2019 Animal cultures matter for conservation. Science **363**, 1032–1034. (10.1126/science.aaw3557) 30808816

[13] Brakes P *et al*. 2021 A deepening understanding of animal culture suggests lessons for conservation. Proc. R. Soc. B **288**, 20202718. (10.1098/rspb.2020.2718) PMC8059593 33878919

[14] Wessling E *et al*. 2025 Concerted conservation actions to support chimpanzee cultures. Phil. Trans. R. Soc. B **380**, 20240143. (10.1098/rstb.2024.0143) 40308136 PMC12044366

[15] Plumptre AJ *et al*. 2010 Eastern chimpanzee (Pan troglodytes schweinfurthii): status survey and conservation action plan 2010–2020. Gland, Switzerland: IUCN.

[16] IUCN SSC Primate Specialist Group. 2020 Regional action plan for the conservation of western chimpanzees (Pan troglodytes verus) 2020–2030. Gland, Switzerland: IUCN.

[17] Kendal RL, Galef BG, van Schaik CP. 2010 Social learning research outside the laboratory: how and why? Learn. Behav. **38**, 187–194. (10.3758/lb.38.3.187) 20628158

[18] Byrne RW, Russon AE. 1998 Learning by imitation: a hierarchical approach. Behav. Brain Sci. **21**, 667–721. (10.1017/s0140525x98001745) 10097023

[19] Whiten A, McGuigan N, Marshall-Pescini S, Hopper LM. 2009 Emulation, imitation, over-imitation and the scope of culture for child and chimpanzee. Phil. Trans. R. Soc. B **364**, 2417–2428. (10.1098/rstb.2009.0069) 19620112 PMC2865074

[20] Fisher J, Hinde R. 1949 The opening of milk bottles by birds. Br. Birds **42**, 347–357.

[21] Cavalli-Sforza L, Feldman K. 1981 Cultural transmission and evolution: a quantitative approach. Princeton, NJ: Princeton University Press. (10.1515/9780691209357) 7300842

[22] Lefebvre L. 1995 Culturally-transmitted feeding behaviour in primates: evidence for accelerating learning rates. Primates **36**, 227–239. (10.1007/bf02381348)

[23] Reader SM. 2004 Distinguishing social and asocial learning using diffusion dynamics. Learn. Behav. **32**, 90–104. (10.3758/bf03196010) 15161144

[24] Franz M, Nunn CL. 2009 Network-based diffusion analysis: a new method for detecting social learning. Proc. R. Soc. B **276**, 1829–1836. (10.1098/rspb.2008.1824) PMC2674490 19324789

[25] Hoppitt W, Boogert NJ, Laland KN. 2010 Detecting social transmission in networks. J. Theor. Biol. **263**, 544–555. (10.1016/j.jtbi.2010.01.004) 20064530

[26] Allen J, Weinrich M, Hoppitt W, Rendell L. 2013 Network-based diffusion analysis reveals cultural transmission of lobtail feeding in humpback whales. Science **340**, 485–488. (10.1126/science.1231976) 23620054

[27] Hobaiter C, Poisot T, Zuberbühler K, Hoppitt W, Gruber T. 2014 Social network analysis shows direct evidence for social transmission of tool use in wild chimpanzees. PLoS Biol. **12**, e1001960. (10.1371/journal.pbio.1001960) 25268798 PMC4181963

[28] Hoppitt W, Farine D. 2018 Association indices for quantifying social relationships: how to deal with missing observations of individuals or groups. Anim. Behav **136**, 227–238. (10.1016/j.anbehav.2017.08.029)

[29] Hoppitt W. 2017 The conceptual foundations of network-based diffusion analysis: choosing networks and interpreting results. Phil. Trans. R. Soc. B **372**, 20160418. (10.1098/rstb.2016.0418) 29061891 PMC5665806

[30] Hasenjager MA, Leadbeater E, Hoppitt W. 2021 Detecting and quantifying social transmission using network‐based diffusion analysis. J. Anim. Ecol. **90**, 8–26. (10.1111/1365-2656.13307) 32745269

[31] Rutz C *et al*. 2016 Discovery of species-wide tool use in the Hawaiian crow. Nature **537**, 403–407. (10.1038/nature19103) 27629645

[32] Cranmer K, Brehmer J, Louppe G. 2020 The frontier of simulation-based inference. Proc. Natl Acad. Sci. USA **117**, 30055–30062. (10.1073/pnas.1912789117) 32471948 PMC7720103

[33] Kandler A, Powell A. 2018 Generative inference for cultural evolution. Phil. Trans. R. Soc. B **373**, 20170056. (10.1098/rstb.2017.0056) 29440522 PMC5812969

[34] Youngblood M, Lahti DC. 2022 Content bias in the cultural evolution of house finch song. Anim. Behav. **185**, 37–48. (10.1016/j.anbehav.2021.12.012)

[35] Whiten A, Mesoudi A. 2008 Establishing an experimental science of culture: animal social diffusion experiments. Phil. Trans. R. Soc. B **363**, 3477–3488. (10.1098/rstb.2008.0134) 18799418 PMC2607342

[36] Whiten A, Caldwell C, Mesoudi A. 2016 Cultural diffusion in humans and other animals. Curr. Opin. Psychol **8**, 15–21. (10.1016/j.copsyc.2015.09.002) 29506791

[37] Whiten A, Spiteri A, Horner V, Bonnie KE, Lambeth SP, Schapiro SJ, de Waal FBM. 2007 Transmission of multiple traditions within and between chimpanzee groups. Curr. Biol. **17**, 1038–1043. (10.1016/j.cub.2007.05.031) 17555968

[38] Bandini E, Motes-Rodrigo A, Steele MP, Rutz C, Tennie C. 2020 Examining the mechanisms underlying the acquisition of animal tool behaviour. Biol. Lett. **16**, 20200122. (10.1098/rsbl.2020.0122) 32486940 PMC7336849

[39] Tennie C, Bandini E, van Schaik CP, Hopper LM. 2020 The zone of latent solutions and its relevance to understanding ape cultures. Biol. Philos. **35**, 55. (10.1007/s10539-020-09769-9) 33093737 PMC7548278

[40] Tennie C. 2023 Focusing on relevant data and correcting misconceptions reaffirms the ape ZLS. Phys. Life Rev. **44**, 94–98. (10.1016/j.plrev.2022.12.007) 36584581

[41] Whiten A. 2022 Blind alleys and fruitful pathways in the comparative study of cultural cognition. Phys. Life Rev. **43**, 211–238. (10.1016/j.plrev.2022.10.003) 36343568

[42] Whiten A. 2024 Diverse species readily acquire copies of novel actions from others that are not achieved through individual learning. Anim. Behav. **216**, 85–96. (10.1016/j.anbehav.2024.08.001)

[43] Aplin LM, Farine DR, Morand-Ferron J, Cockburn A, Thornton A, Sheldon BC. 2015 Experimentally induced innovations lead to persistent culture via conformity in wild birds. Nature **518**, 538–541. (10.1038/nature13998) 25470065 PMC4344839

[44] Canteloup C, Hoppitt W, van de Waal E. 2020 Wild primates copy higher-ranked individuals in a social transmission experiment. Nat. Commun **11**, 459. (10.1038/s41467-019-14209-8) 31974385 PMC6978360

[45] Arbon JJ, Hahn LG, McIvor GE, Thornton A. 2023 Competition and generalization impede cultural formation in wild jackdaws. Proc. R. Soc. B **290**, 20230705. (10.1098/rspb.2023.0705) PMC10410225 37554031

[46] Aplin LM, Sheldon BC, McElreath R. 2017 Conformity does not perpetuate suboptimal traditions in a wild population of songbirds. Proc. Natl Acad. Sci. USA **114**, 7830–7837. (10.1073/pnas.1621067114) 28739943 PMC5544276

[47] McElreath R, Lubell M, Richerson PJ, Waring TM, Baum W, Edsten E, Efferson C, Paciotti B. 2005 Applying evolutionary models to the laboratory study of social learning. Evol. Hum. Behav. **26**, 483–508. (10.1016/j.evolhumbehav.2005.04.003)

[48] McElreath R, Bell AV, Efferson C, Lubell M, Richerson PJ, Waring T. 2008 Beyond existence and aiming outside the laboratory: estimating frequency-dependent and pay-off-biased social learning strategies. Phil. Trans. R. Soc. B **363**, 3515–3528. (10.1098/rstb.2008.0131) 18799416 PMC2607339

[49] Barrett BJ, McElreath RL, Perry SE. 2017 Pay-off-biased social learning underlies the diffusion of novel extractive foraging traditions in a wild primate. Proc. R. Soc. B **284**, 20170358. (10.1098/rspb.2017.0358) PMC5474070 28592681

[50] Schuppli C, van Schaik CP. 2019 Animal cultures: how we’ve only seen the tip of the iceberg. Evol. Hum. Sci. **1**, e2. (10.1017/ehs.2019.1) 37588402 PMC10427297

[51] Whiten A, Goodall J, McGrew WC, Nishida T, Reynolds V, Sugiyama Y, Tutin CEG, Wrangham RW, Boesch C. 2001 Charting cultural variation in chimpanzees. Behaviour **13**, 1489–1525. (10.1163/156853901317367717)

[52] Laland K, Janik V. 2006 The animal cultures debate. Trends Ecol. Evol. **21**, 542–547. (10.1016/j.tree.2006.06.005) 16806574

[53] Krützen M, Whiten A, van Schaik C. 2007 The animal cultures debate: response to Laland and Janik. Trends Ecol. Evol **22**, 6. (10.1016/j.tree.2006.10.011) 17088010

[54] Laland K, Galef B (eds). 2009 The question of animal culture. Cambridge, MA: Harvard University Press. (10.2307/j.ctv322v4wf)

[55] Krützen M, Willems EP, van Schaik CP. 2011 Culture and geographic variation in orangutan behavior. Curr. Biol. **21**, 1808–1812. (10.1016/j.cub.2011.09.017) 22018539

[56] Boesch C *et al*. 2020 Chimpanzee ethnography reveals unexpected cultural diversity. Nat. Hum. Behav. **4**, 910–916. (10.1038/s41562-020-0890-1) 32451479

[57] Wild S, Hoppitt WJE, Allen SJ, Krützen M. 2020 Integrating genetic, environmental, and social networks to reveal transmission pathways of a dolphin foraging innovation. Curr. Biol. **30**, 3024–3030.(10.1016/j.cub.2020.05.069) 32589911

[58] Humle T, Matsuzawa T. 2002 Ant‐dipping among the chimpanzees of Bossou, Guinea, and some comparisons with other sites. Am. J. Primatol. **58**, 133–148. (10.1002/ajp.10055) 12454957

[59] Rutz C, Hunt GR, St Clair JJH. 2018 Corvid technologies: how do New Caledonian crows get their tool designs? Curr. Biol. **28**, R1109–R1111. (10.1016/j.cub.2018.08.031) 30253153

[60] Dindo M, Stoinski T, Whiten A. 2011 Observational learning in orangutan cultural transmission chains. Biol. Lett. **7**, 181–183. (10.1098/rsbl.2010.0637) 20843841 PMC3061153

[61] Hunt GR, Gray RD. 2003 Diversification and cumulative evolution in New Caledonian crow tool manufacture. Proc. R. Soc. B **270**, 867–874. (10.1098/rspb.2002.2302) PMC1691310 12737666

[62] Catchpole C, Slater P. 2008 Bird song: biological themes and variations, 2nd edn. Cambridge, UK: Cambridge University Press.

[63] Geller FC, Lahti DC. 2023 Is sexiness cumulative? Arguments from birdsong culture. Anim. Behav. **205**, 131–137. (10.1016/j.anbehav.2023.09.006)

[64] Garland EC, McGregor PK. 2020 Cultural transmission, evolution, and revolution in vocal displays: insights from bird and whale song. Front. Psychol. **11**, 544929. (10.3389/fpsyg.2020.544929) 33132953 PMC7550662

[65] Garland E *et al*. 2025 Culture and conservation in baleen whales. Phil. Trans. R. Soc. B **380**, 20240133. (10.1098/2024.0133) 40308135 PMC12044387

[66] Luncz LV, Mundry R, Boesch C. 2012 Evidence for cultural differences between neighbouring chimpanzee (Pan troglodytes verus) communities. Curr. Biol. **22**, 922–926. (10.1016/j.cub.2012.03.031) 22578420

[67] van Leeuwen E, Cronin K, Haun S. 2014 A group specific arbitrary tradition in chimpanzees. Anim. Cogn **17**, 1421–1425. (10.1007/s10071-014-0766-8) 24916739

[68] Goodall J. 1973 Cultural elements in a chimpanzee community. In Precultural primate behaviour (ed E Menzel), pp. 144–184. Basel, Switzerland: Karger.

[69] Jaeggi AV, Dunkel LP, van Noordwijk MA, Wich SA, Sura AAL, van Schaik CP. 2010 Social learning of diet and foraging skills by wild immature Bornean orangutans: implications for culture. Am. J. Primatol. **72**, 62–71. (10.1002/ajp.20752) 19790189

[70] Jaeggi AV, van Noordwijk MA, van Schaik CP. 2008 Begging for information: mother–offspring food sharing among wild Bornean orangutans. Am. J. Primatol. **70**, 533–541. (10.1002/ajp.20525) 18186082

[71] Schuppli C, Meulman EJM, Forss SIF, Aprilinayati F, van Noordwijk MA, van Schaik CP. 2016 Observational social learning and socially induced practice of routine skills in immature wild orang-utans. Anim. Behav. **119**, 87–98. (10.1016/j.anbehav.2016.06.014)

[72] Ehmann B, van Schaik CP, Ashbury AM, Mörchen J, Musdarlia H, Utami Atmoko S, van Noordwijk MA, Schuppli C. 2021 Immature wild orangutans acquire relevant ecological knowledge through sex-specific attentional biases during social learning. PLoS Biol. **19**, e3001173. (10.1371/journal.pbio.3001173) 34010339 PMC8133475

[73] Mörchen J *et al*. 2023 Migrant orangutan males use social information to adapt to new habitat after dispersal. Front. Ecol. Evol **11**, 1158887. (10.3389/fevo.2023.1158887)

[74] Lonsdorf EV, Eberly LE, Pusey AE. 2004 Sex differences in learning in chimpanzees. Nature **428**, 715–716. (10.1038/428715a) 15085121

[75] Müller CA, Cant MA. 2010 Imitation and traditions in wild banded mongooses. Curr. Biol. **20**, 1171–1175. (10.1016/j.cub.2010.04.037) 20605459

[76] Slagsvold T, Wiebe KL. 2011 Social learning in birds and its role in shaping a foraging niche. Phil. Trans. R. Soc. B **366**, 969–977. (10.1098/rstb.2010.0343) 21357219 PMC3049099

[77] Thornton A, McAuliffe K. 2006 Teaching in wild meerkats. Science **313**, 227–229. (10.1126/science.1128727) 16840701

[78] Hoppitt WJE, Brown GR, Kendal R, Rendell L, Thornton A, Webster MM, Laland KN. 2008 Lessons from animal teaching. Trends Ecol. Evol. **23**, 486–493. (10.1016/j.tree.2008.05.008) 18657877

[79] Thornton A, Raihani N. 2008 The evolution of teaching. Anim. Behav **75**, 1823–1836. (10.1016/j.anbehav.2007.12.014)

[80] Musgrave S, Morgan D, Lonsdorf E, Mundry R, Sanz C. 2016 Tool transfers are a form of teaching in chimpanzees. Sci. Rep **6**, 34783. (10.1038/srep34783) 27725706 PMC5057084

[81] Musgrave S, Lonsdorf E, Morgan D, Prestipino M, Bernstein-Kurtycz L, Mundry R, Sanz C. 2020 Teaching varies with task complexity in wild chimpanzees. Proc. Natl Acad. Sci. USA **117**, 969–976. (10.1073/pnas.1907476116) 31871167 PMC6969499

[82] Mercader J, Barton H, Gillespie J, Harris J, Kuhn S, Tyler R, Boesch C. 2007 4,300-year-old chimpanzee sites and the origins of percussive stone technology. Proc. Natl Acad. Sci. USA **104**, 3043–3048. (10.1073/pnas.0607909104) 17360606 PMC1805589

[83] Falótico T, Proffitt T, Ottoni EB, Staff RA, Haslam M. 2019 Three thousand years of wild capuchin stone tool use. Nat. Ecol. Evol. **3**, 1034–1038. (10.1038/s41559-019-0904-4) 31235926

[84] Pascual-Garrido A, Carvalho S, Almeida-Warren K. 2023 Primate archaeology 3.0. Am. J. Biol. Anthropol. **183**, e24835. (10.1002/ajpa.24835) 37671610

[85] Pascual-Garrido A. 2019 Cultural variation between neighbouring communities of chimpanzees at Gombe, Tanzania. Sci. Rep. **9**, 8260. (10.1038/s41598-019-44703-4) 31164683 PMC6547654

[86] Mesoudi A, Thornton A. 2018 What is cumulative cultural evolution? Proc. R. Soc. B **285**, 20180712. (10.1098/rspb.2018.0712) PMC6015846 29899071

[87] McElreath MB, Boesch C, Kühl H, McElreath R. 2018 Complex dynamics from simple cognition: the primary ratchet effect in animal culture. Evol. Behav. Sci. **12**, 191–202. (10.1037/ebs0000117)

[88] Gruber T, Chimento M, Aplin LM, Biro D. 2022 Efficiency fosters cumulative culture across species. Phil. Trans. R. Soc. B **377**, 20200308. (10.1098/rstb.2020.0308) 34894729 PMC8666915

[89] Henrich J. 2015 The secret of our success: how culture is driving human evolution, domesticating our species and making us smarter. Princeton, NJ: Princeton University Press.

[90] Rawlings BS, Legare CH, Brosnan SF, Vale GL. 2021 Leveling the playing field in studying cumulative cultural evolution: conceptual and methodological advances in nonhuman animal research. J. Exp. Psychol. Animal Learn. Cog. **47**, 252–273. (10.1037/xan0000303) 34618526

[91] Greggor A *et al*. 2025 Strategies for integrating social learning into conservation translocation practice. Phil. Trans. R. Soc. B **380**, 20240138. (10.1098/rstb.2024.0138) 40308141 PMC12044373

[92] Jesmer BR *et al*. 2018 Is ungulate migration culturally transmitted? Evidence of social learning from translocated animals. Science **361**, 1023–1025. (10.1126/science.aat0985) 30190405

[93] Hunt GR. 1996 Manufacture and use of hook-tools by New Caledonian crows. Nature **379**, 249–251. (10.1038/379249a0)

[94] Rutz C, Hunt GR. 2020 New Caledonian crows afford invaluable comparative insights into human cumulative technological culture. Behav. Brain Sci. **43**, E177. (10.1017/s0140525x20000187) 32772983

[95] St Clair JJH, Klump BC, Sugasawa S, Higgott CG, Colegrave N, Rutz C. 2018 Hook innovation boosts foraging efficiency in tool-using crows. Nat. Ecol. Evol. **2**, 441–444. (10.1038/s41559-017-0429-7) 29358606

[96] St Clair JJH, Rutz C. 2013 New Caledonian crows attend to multiple functional properties of complex tools. Phil. Trans. R. Soc. B **368**, 20120415. (10.1098/rstb.2012.0415) 24101625 PMC4027419

[97] Williams H, Levin II, Norris DR, Newman AEM, Wheelwright NT. 2013 Three decades of cultural evolution in savannah sparrow songs. Anim. Behav. **85**, 213–223. (10.1016/j.anbehav.2012.10.028)

[98] Williams H, Lachlan RF. 2022 Evidence for cumulative cultural evolution in bird song. Phil. Trans. R. Soc. B **377**, 20200322. (10.1098/rstb.2020.0322) 34894731 PMC8666912

[99] Allen JA, Garland EC, Dunlop RA, Noad MJ. 2018 Cultural revolutions reduce complexity in the songs of humpback whales. Proc. R. Soc. B **285**, 20182088. (10.1098/rspb.2018.2088) PMC6253384 30464066

[100] Delisle ZJ, Flaherty EA, Nobbe MR, Wzientek CM, Swihart RK. 2021 Next-generation camera trapping: systematic review of historic trends suggests keys to expanded research applications in ecology and conservation. Front. Ecol. Evol. **9**, 617996. (10.3389/fevo.2021.617996)

[101] Kays R, Crofoot MC, Jetz W, Wikelski M. 2015 Terrestrial animal tracking as an eye on life and planet. Science **348**, aaa2478. (10.1126/science.aaa2478) 26068858

[102] Hussey NE *et al*. 2015 Aquatic animal telemetry: a panoramic window into the underwater world. Science **348**, 1255642. (10.1126/science.1255642) 26068859

[103] Sakamoto KQ, Sato K, Ishizuka M, Watanuki Y, Takahashi A, Daunt F, Wanless S. 2009 Can ethograms be automatically generated using body acceleration data from free-ranging birds? PLoS One **4**, e5379. (10.1371/journal.pone.0005379) 19404389 PMC2671159

[104] Adachi T *et al*. 2021 Forced into an ecological corner: round-the-clock deep foraging on small prey by elephant seals. Sci. Adv. **7**, eabg3628. (10.1126/sciadv.abg3628) 33980496 PMC8115928

[105] Tanigaki K, Otsuka R, Li A, Hatano Y, Wei Y, Koyama S, Yoda K, Maekawa T. 2024 Automatic recording of rare behaviors of wild animals using video bio-loggers with on-board light-weight outlier detector. PNAS Nexus **3**, pgad447. (10.1093/pnasnexus/pgad447) 38229952 PMC10791039

[106] Cliff OM, Saunders DL, Fitch R. 2018 Robotic ecology: tracking small dynamic animals with an autonomous aerial vehicle. Sci. Robot. **3**, eaat8409. (10.1126/scirobotics.aat8409) 33141732

[107] Mueller T, O’Hara RB, Converse SJ, Urbanek RP, Fagan WF. 2013 Social learning of migratory performance. Science **341**, 999–1002. (10.1126/science.1237139) 23990559

[108] Bodey TW, Cleasby IR, Bell F, Parr N, Schultz A, Votier SC, Bearhop S. 2018 A phylogenetically controlled meta‐analysis of biologging device effects on birds: deleterious effects and a call for more standardized reporting of study data. Methods Ecol. Evol. **9**, 946–955. (10.1111/2041-210x.12934)

[109] Parnell AC *et al*. 2013 Bayesian stable isotope mixing models. Environmetrics **24**, 387–399. (10.1002/env.2221)

[110] Ando H, Mukai H, Komura T, Dewi T, Ando M, Isagi Y. 2020 Methodological trends and perspectives of animal dietary studies by noninvasive fecal DNA metabarcoding. Environ. DNA **2**, 391–406. (10.1002/edn3.117)

[111] Gunasekaram C *et al*. 2024 Population connectivity shapes the distribution and complexity of chimpanzee cumulative culture. Science **386**, 920–925. (10.1126/science.adk3381) 39571020

[112] Dussex N *et al*. 2021 A genome‐wide investigation of adaptive signatures in protein‐coding genes related to tool behaviour in New Caledonian and Hawaiian crows. Mol. Ecol. **30**, 973–986. (10.1111/mec.15775) 33305388

[113] Tuia D *et al*. 2022 Perspectives in machine learning for wildlife conservation. Nat. Commun **13**, 792. (10.1038/s41467-022-27980-y) 35140206 PMC8828720

[114] Rutz C, Bronstein M, Raskin A, Vernes SC, Zacarian K, Blasi DE. 2023 Using machine learning to decode animal communication. Science **381**, 152–155. (10.1126/science.adg7314) 37440653

[115] Schofield D, Nagrani A, Zisserman A, Hayashi M, Matsuzawa T, Biro D, Carvalho S. 2019 Chimpanzee face recognition from videos in the wild using deep learning. Sci. Adv. **5**, eaaw0736. (10.1126/sciadv.aaw0736) 31517043 PMC6726454

[116] Bain M *et al*. 2021 Automated audiovisual behavior recognition in wild primates. Sci. Adv. **7**, eabi4883. (10.1126/sciadv.abi4883) 34767448 PMC8589313

[117] Wiltshire C, Lewis‐Cheetham J, Komedová V, Matsuzawa T, Graham KE, Hobaiter C. 2023 DeepWild: application of the pose estimation tool DeepLabCut for behaviour tracking in wild chimpanzees and bonobos. J. Anim. Ecol. **92**, 1560–1574. (10.1111/1365-2656.13932) 37165474

[118] Dezecache G, Zuberbühler K, Davila-Ross M, Dahl CD. 2021 A machine learning approach to infant distress calls and maternal behaviour of wild chimpanzees. Anim. Cogn. **24**, 443–455. (10.1007/s10071-020-01437-5) 33094407

[119] Kalan AK *et al*. 2020 Environmental variability supports chimpanzee behavioural diversity. Nat. Commun. **11**, 4451. (10.1038/s41467-020-18176-3) 32934202 PMC7493986

[120] Kühl HS *et al*. 2019 Human impact erodes chimpanzee behavioral diversity. Science **363**, 1453–1455. (10.1126/science.aau4532) 30846610

[121] Bessa J, Biro D, Hockings K. 2022 Inter-community behavioural variation confirmed through indirect methods in four neighbouring chimpanzee communities in Cantanhez NP, Guinea-Bissau. R. Soc. Open Sci. **9**, 211518. (10.1098/rsos.211518) 35223060 PMC8864369

[122] Bluff LA, Kacelnik A, Rutz C. 2010 Vocal culture in New Caledonian crows Corvus moneduloides. Biol. J. Linn. Soc. **101**, 767–776. (10.1111/j.1095-8312.2010.01527.x)

[123] Foote AD *et al*. 2016 Genome-culture coevolution promotes rapid divergence of killer whale ecotypes. Nat. Commun. **7**, 11693. (10.1038/ncomms11693) 27243207 PMC4895049

[124] St Clair JJH, Burns ZT, Bettaney EM, Morrissey MB, Otis B, Ryder TB, Fleischer RC, James R, Rutz C. 2015 Experimental resource pulses influence social-network dynamics and the potential for information flow in tool-using crows. Nat. Commun. **6**, 7197. (10.1038/ncomms8197) 26529116 PMC4659832

[125] Webster MM, Rutz C. 2020 How STRANGE are your study animals? Nature **582**, 337–340. (10.1038/d41586-020-01751-5) 32541916

[126] Whiten A. 2017 Culture extends the scope of evolutionary biology in the great apes. Proc. Natl Acad. Sci. USA **114**, 7790–7797. (10.1073/pnas.1620733114) 28739927 PMC5544264

[127] Brakes P, Aplin L, Carroll EL, Greggor AL, Whiten A, Garland EC. 2025 Animal culture: conservation in a changing world. Phil. Trans. R. Soc. B **380**, 20240127. (10.1098/rstb.2024.0127) 40308140 PMC12044377

[128] Whiten A, Rutz C. 2025 Supplementary material from: The growing methodological toolkit for identifying and studying social learning and culture in non-human animals. Figshare. (10.6084/m9.figshare.c.7775096) PMC12044376 40308147

